# Age and education as factors associated with medication literacy: a community pharmacy perspective

**DOI:** 10.1186/s12877-020-01881-5

**Published:** 2020-11-25

**Authors:** Javier Plaza-Zamora, Isabel Legaz, Eduardo Osuna, María D. Pérez-Cárceles

**Affiliations:** 1 Community Pharmacy, Health Service of Murcia, Murcia, Spain; 2 Department of Legal and Forensic Medicine, Institute of Research into Aging. Biomedical Research Institute (IMIB), Regional Campus of International Excellence “Campus Mare Nostrum”, Faculty of Medicine, University of Murcia, Murcia, Spain

**Keywords:** Aging, Community pharmacy, Education, Legal medicine, Patient safety

## Abstract

**Background:**

Aging implies a higher prevalence of chronic pathologies and a corresponding increase in medication. The correct adherence and use of the medication are prerequisites for reducing risks of disease progression, comorbidity, and mortality. Medication literacy (ML) is the specific ability to safely access and understand the information available concerning medication, and to act accordingly. Currently, there are few specific instruments that ascertain the extent of ML in the general population. The aim of this work was to analyse ML in a large cohort of pharmacy customers.

**Methods:**

A total of 400 community pharmacy clients were analyzed to assess the level of ML (documental and numeracy) through the validated MedLitRxSE tool.

**Results:**

The results showed that out of a total of 400 community pharmacy clients only 136 (34%) had an adequate degree of ML, while the rest of the clients (*n* = 264; 66%) were adjudged not to have this ability. Statistically significant differences were found between the different age groups in terms of ML (*P* < 0.001; OR = 0.312; 95% CI: 0.195–0.499), the 51–65 and >65-year age groups having a lower frequency of adequate ML (23.5 and 7.1%, respectively) than the rest of the age groups. A statistically significant increase in adequate ML was observed as the academic level of the clients increased (*P* < 0.001; OR = 15.403; 95% CI: 8.109–29.257). Multivariate logistic regression confirmed the influence of both variables on ML.

**Conclusions:**

An inadequate ML level was found in community pharmacy clients over the age of 51, and also in those with primary or non-formal studies. Our data add to our knowledge about ML, and should pharmacists and other health professionals to adopt new strategies to prevent, or at least reduce, errors in taking medicines, thus avoiding the undesirable effects of any misuse.

**Supplementary Information:**

The online version contains supplementary material available at 10.1186/s12877-020-01881-5.

## Background

Aging implies a higher prevalence of chronic pathologies and therefore an increase in medication [1, 2]. However, the correct use of the medication is a prerequisite for reducing the risks of disease progression, comorbidity, and mortality [3, 4].

Older patients are major users of both prescription and non-prescription medicines, and their proper use will lead to more cost-effective approaches and increase safety [5–7]. Medication non-adherence, or the extent to which patients do not take their medications as agreed with their health care provider, averages 50% among patients suffering from chronic diseases in developed countries [5] and results in poorer health outcomes and a lower quality of life in patients [8]. Inadequate prescription details or misunderstanding instructions is often associated with limited information and a poor knowledge of medication use, leading to errors and less effective treatment [9, 10]. Moreover, most adverse drug reactions are related to excessive dosage and drug interactions associated with polypharmacy [11, 12].

Medication literacy (ML) is the specific ability to safely access and understand the information available concerning medication, and act accordingly [13]. Although there are some instruments available for its evaluation [13], ML is not fully recognized. Only about 50% of patients [11] follow chronic medication instructions, a figure that could be improved by encouraging the participation of patients in their treatments. In order to achieve the maximum effect and a safety in the use of medication, patients and caregivers must have adequate knowledge of the therapy. Perhaps more seriously, the safety of children is at risk due to parents’ lack of knowledge about medication administration [9, 14, 15].

A proper use of the medication includes how to administer it, when, for how long, what quantity [16]. This information must be provided first by the health professionals in a simple and effective way adapted to the particular patient but also by medication information sheets that are readable with pictograms to help patient comprehension and avoid the misuse of the medicines [17, 18]. For their part, pharmacists want their patients to take their medication and to follow treatment strategy properly. To meet these standards, it is critical that patients not only have sufficient information, but also sufficient reading, coding, and self-management skills to use that information [19, 20].

ML includes skills such as interpretation and the ability to calculate doses, and cannot be measured properly by general evaluations of literacy. However, it is important to be able to assess numerical and/or documental knowledge before receiving written or verbal instructions from health professionals about preparing medication doses, the duration of treatment and any warnings [21]. Community pharmacists could evaluate ML prior to carrying out a medication review [22], so they can know what areas need reinforcing to improve the proper understanding by the patient, thus influencing their ability to adhere to the treatment. Despite the absence of tools to measure medical literacy, there is a need on the part of community pharmacists to quickly and specifically assess the ML of their patients.

In this study the ML of clients acquiring prescription and non-prescription medicines in community pharmacies was analyzed using MedlitRxSE questionnaire in order to the future purpose of designing new strategies that allow the pharmacist and health personnel to prevent and reduce the errors in taking medicines and thus avoid the undesirable effects of any misuse.

## Methods

### Participants

The sample size initially calculated was to represent a population of 2500 people, since in Spain this is the average number of inhabitants per pharmacy office, with an accuracy of 5% (e = 0.05) and with a 95% confidence interval. The calculated sample size was 334 subjects, to which we added 20% to cover possible withdrawals. The final sample consisted of 400 community pharmacy clients.

All 400 clients answered the validated MedLitRxSE tool [13] anonymously. The pharmacy staff from each pharmacy office participating provided information about the study when they went to the pharmacy office. The data for this study were collected at the pharmacy office. The clients comprised caregivers or clients who simply came to pick up collect medication for their relatives or for themselves.

Four community pharmacies were randomly selected from 20 offices in an area in south-eastern Spain. They were located in rural and urban areas. Rural areas were defined as areas with a low population density (< 2500 habitants) and urban areas were those with a high population density (>2500 inhabitants) [23]. Each pharmacy conducted 100 surveys, which included sociodemographic data, the consumption of medication and the frequency with which the patient read the information leaflet. The study was carried out between January 2016 and December 2018.

The inclusion criteria considered were clients over 18 years of age, users of public or private health services who went to the community pharmacy and asked for a medicine prescribed by a doctor or an over-the-counter medicine for personal use or for someone else.

All participants were informed of the study through the pharmaceutical staff attached to the community pharmacy, obtaining written informed consent in all cases. The study and protocols for recruitment were approved by the Human Research Ethics Committee of the University of Murcia (Approval number: 1896/2018, date of approval 10 April 2018) in accordance with the ‘Ethical Principles for Medical Research Involving Human Subjects’ adopted in the Helsinki Declaration by the World Medical Association (64thth WMA General Assembly, Fortaleza, Brazil, October 2013).

### Medication literacy assessment

To use the MedLitRxSEs (Medication Literacy Assessment) tool, the authors were previously contacted, and they provided us with all the necessary information. A psychometric evaluation of this new assessment tool is described by authors who designed the instrument, and the Spanish version has been shown satisfactory in this respect (13). The MedLitRxSE is a tool that assesses the skills needed to manage medication properly. It consists of 14 items organized in four scenarios, of which 10 items relate to document literacy and 4 to numeracy, all with a dichotomous response (Table S1). A possible range of 0–14 points can be obtained, in such a way that a higher score reflects greater literacy with medication. The MedLitRxSE does not have scoring criteria from which medication literacy levels can be obtained, but identifying the most commonly missed questions can give pharmacists clues as to the deficiencies of patients in understanding prescription information and dosage instructions. Any error suggests potential confusion or concern about the safety or use of the medication. Thus, pharmacists who provide medication or disease therapy management services may consider including this questionnaire on the first occasion a patient visits the pharmacy as a way of assessing any areas of possible misunderstanding related to the use of medications [13].

In order to understand the factors associated with ML, this qualitative variable (documental and numeracy) was converted into 2 dichotomies: inadequate and adequate ML, and a cut-off point was established to categorize ML. MedLiTRxSE does not establish a cut-off point to differentiate between adequate and inadequate medication literacy. As experts in the field we established that of the 14 possible points in MedLiTRxSE tool with 13 points we should consider an adequate ML. To achieve a higher range (adequate literacy) we assumed that of the 14 competencies evaluated by the tool, only one could fail: knowing the name of the doctor who had prescribed the drug or knowing the name of the active ingredients, both competencies from documentary medication literacy. In the case of numeracy medication literacy, we consider that when trying to know and use the doses of the drugs in an appropriate way, the requirement to consider it as adequate was greater, establishing the four questions as correct. Therefore, an adequate literacy score was established to be ≥13 points for total ML, ≥9 points for document ML, and 4 points for numeracy-ML.

Univariate and multivariate analyses with logistic regression of all the variables pointed to a significant relation with the dependent variable in the bivariate analyses [24]. Then, by backward stepwise selection, a model was obtained with the individual variables directly related to the dependent variable, adequate ML.

### Statistical analysis

Sociodemographic data and the results of each survey were collected in a database (Microsoft Access 11.0; Microsoft corporation, Seattle, WA), and statistical analysis was performed using the SPSS 15.0 software (SPSS Inc., Chicago IL, USA). Categorical data were compared using chi-square (X^2^) test or Fisher’s Exact test, and non-parametric count data were compared using the Kruskal Wallis test or Mann Whitney U test. Inadequate and adequate ML were compared using bivariate analyses to determine whether there was any difference in the descriptive characteristics (age, sex, education). Logistic regression univariate and multivariate analysis was used to examine factors associated with adequate medication literacy. A level of *P* < 0.05 was accepted as statistically significant. The odds ratios (OR) and their 95% confidence intervals (CI) were calculated in order to estimate relative risk. The I-square (I2) statistic with cut-off values of 73.1% and a *p*-value of < 0.001 was considered statistically significant.

## Results

### Demographic characteristics

A total of 400 community pharmacy clients, including 136 males (34%) and 264 females (66%) were analyzed in this study (Table 1). The median age of the total cohort was 49.65 ± 16.62 (years ± SD). The four age groups analyzed were equally represented and no significant differences were found (X^2^ = 0.090). Individuals under 50 years represented 53.5% of the total while individuals over or equal to 51 years represented 46.5%.

**Table 1:** Analysis of the sociodemographic characteristics, pharmacies areas, number of medications consumed and reading information leaflet of community pharmacy clients and its relationship with medicacion literacy. (a) Analysis of adequate or inadequate medication literacy (b) Analysis of correct answers in MedLiTRxSE tool

	Total ***N*** = 400, n (%)	(a) Medication Literacy		P1	(b) Medication Literacy				P3	P4
		Adequate ***N*** = 136, n (%)	Inadequate ***N =*** 264, n (%)		Total	P2	Documental	Numerical		
**Gender**										
Male	136 (34 .0)	57 (41.9)	79 (58.1)	**0.019**^**a**^	10.72 ± 3.52*	0.075	7.42 ± 2.59	3.31 ± 1.08	**0.008**	0.268
Female	264 (66.0)	79 (29.9)	185 (70.1)		10.08 ± 3.34		6.88 ± 2.46	3.23 ± 1.09		
**Age intervals, years**										
< 35	99 (24.8)	54 (54.5)	45 (45.5)		12.28 ± 1.58		8.5 2 ± 1.30	3.77 ± 0.51		
35–50	115 (28.7)	52 (45.2)	63 (54.8)	**< 0.001**^**b**^	11.50 ± 2.33	**< 0.001**	7.89 ± 1.87	3.61 ± 0.66	**< 0.001**	**< 0.001**
51–65	102 (25.5)	24 (23.5)	78 (76.5)		10.00 ± 3.09		6.81 ± 2.31	3.19 ± 0.99		
> 65	84 (21.0)	6 (7.1)	78 (92.9)		6.68 ± 3.73		4.54 ± 2.66	2.14 ± 1.35		
**Education level**										
Primary or no formal studies or any study	170 (42.5)	12 (7.1)	158 (92.9)		4.77 ± 3.42		2.91 ± 2.45	1.86 ± 1.17		
Secondary	129 (32.3)	57 (44.2)	72 (55.8)	**< 0.001**^**c**^	11.80 ± 1.81	**< 0.001**	8.10 ± 1.56	3.70 ± 0.54	**< 0.001**	**< 0.001**
University	101 (25.2)	67 (66.3)	34 (33.7)		12.67 ± 1.46		8.79 ± 1.28	3.88 ± 0.35		
**Pharmacies areas**										
Rural	164 (41.0)	64 (39.0)	100 (61.0)	0.086	10.65 ± 3.36	0.083	7.32 ± 2.45	3.33 ± 1.10	0.069	0.154
Urban	236 (59.0)	72 (30.5)	164 (69.5)		10.05 ± 3.43		6.89 ± 2.54	3.17 ± 1.08		
**Number of medications consumed**										
No medicines	151 (37.8)	74 (49.0)	77 (51.0)	**< 0.001**^**d**^	11.81 ± 2.17	**< 0.001**	8.15 ± 1.67	3.67 ± 0.68	**< 0.001**	**< 0.001**
1–4 medicines	179 (44.7)	57 (31.8)	122 (68.2)		10.31 ± 3.21		7.06 ± 2.44	3.26 ± 0.99		
≥ 5 medicines	70 (17.5)	5 (7.1)	65 (92.9)		6.99 ± 3.78		4.75 ± 2.67	2.23 ± 1.36		
**Consumption of chronic medication**										
Yes	249 (44.8)	62 (24.9)	187 (75.1)	**< 0.001**^**e**^	6.08 ± 3.16	**< 0.001**	6.03 ± 2.18	2.86 ± 1.34	**0.002**	**0.002**
Non	151 (37.7)	74 (49.0)	77 (51.0)		10.91 ± 2.84		7.56 ± 2.05	3.36 ± 1.01		
**Reading information leaflet**										
Never	99 (24.8)	28 (28.3)	71 (71.7)		8.94 ± 4.32		6.08 ± 3.16	2.86 ± 1.34		
Sometimes	113 (28.2)	40 (35.4)	73 (64.6)	0.180	10.47 ± 3.05	**0.004**	7.11 ± 2.34	3.35 ± 0.88	**0.002**	**0.002**
Always	188 (47.0)	68 (36.2)	120 (63.8)		10.91 ± 2.84		7.58 ± 2.37	3.36 ± 1.01		

*N* total number of individuals, *n* number of individuals in each study, *SD* standard deviation. P1. Comparisons adequate and inadequate were made by the two-sided Fisher’s exact test or Pearson’s Chi-Square test respectively. P2–4. Comparisons were made by the Mann-Whitney U test or the Kruskal-Wallis test. *P*-values marked in bold are statistically significant (*P <* 0.05). P1. *P*-value obtained comparing inadequate medication literacy group versus adequate medication literacy in all groups. OR. odds ratio with a confidence interval (CI) of 95%.

^a.^OR = 0.592; 95% CI:0.385–0.910. *P =* 0.019 (*P-*value obtained comparing male versus female)

^b.^OR = 0.312; 95% CI: 0.195–0.499. *P <* 0.001 (*P-*value obtained comparing 18–35 years’ group versus the rest of the groups

^c.^OR = 15.403; 95% CI: 8.109–29.257. *P* < 0.001 (*P-*value obtained comparing primary level versus the rest of the groups)

^d.^OR = 2.899; 95% CI: 1.887–4.453. *P <* 0.001 (*P-*value obtained comparing no medicines consumption versus the rest of the groups)

^**e,**^**OR = 0.345; 95% CI: 0.225–0.530,** ***P <*** **0.001** (*P*-value obtained comparing consumption of chronic medication versus non consumption *All values are expressed as mean ± SD

Analysis of the different education levels pointed to statistically significant (X^2^ = 0.003) differences: 42.5% had received only primary education or no official education, and 57.5% had secondary or university levels of education (32.3 and 25.2%, respectively).

As regards the pharmacies where the clients’ replies were analyzed, 41% were rural community pharmacies, while 59% were in urban areas. Finally, as regards the reading of medicine leaflets, 24.8% said they never read the information provided, 28.2% sometimes and 47.0% always. There was no significant association between adequate and inadequate literacy and leaflet reading frequency (*P* = 0.180). However, an increase in the frequency of reading the leaflets did lead to a significantly higher score for mean total medication and documentary literacy, and numeracy.

### Analysis of adequate or inadequate medication literacy

**Fig. 1:**
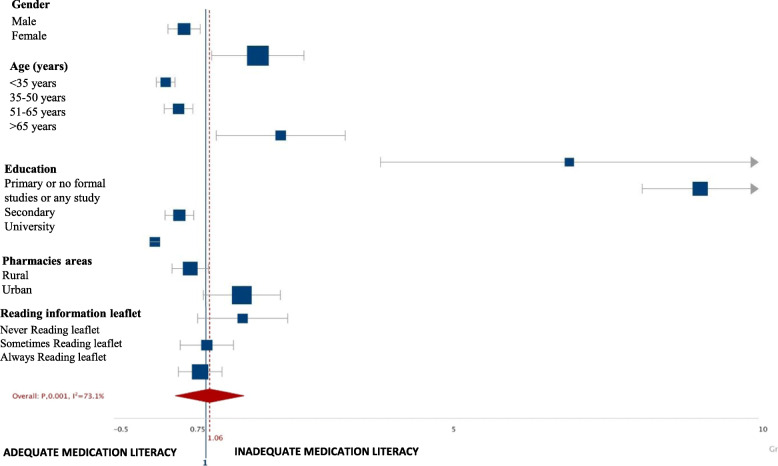
Medication literacy comparing adequate or inadequate literacy in community pharmacy clients with different sociodemographic characteristics. The squares and horizontal lines correspond to the study-specific OR and 95% CI. The area of the squares reflects the weight (inverse of the variance). The diamond represents the OR and 95% CI. Odds Ratios higher than 1 indicate the existence of a causal relationship between the sociodemographic variable analysed and inadequate medication literacy. The results are presented as Odds Ratio with a confidence interval (CI) of 95%. OR; Odds ratio; LCL; Lower confidence level, UCL; Upper confidence level

When sociodemographic characteristics and adequate- or inadequate ML were analyzed (Table 1, Fig. 1), the results showed only 136 clients (34%) had an adequate degree of ML, while the rest were seen to have inadequate ML (*n* = 264; 66%).

More men than women were adjudged to have and adequate level of ML (41.9 and 29.9%, respectively; *P* = 0.019, OR = 0.592, 95% CI: 0.385–0.910). The analysis found statistically significant differences between the different age groups in terms of ML (*P* < 0.001; OR = 0.312; 95% CI: 0.195–0.499), the 51–65 and >65-year age groups having a lower frequency of adequate ML (23.5 and 7.1%, respectively) than the rest of the age groups. A statistically significant increase in adequate ML was observed as the academic level of the clients increased (*P* < 0.001; OR = 15.403; 95% CI: 8.109–29.257).

Similar results concerning adequate ML were found for the rural and urban pharmacies (39 and 30.5% respectively, *P* = 0.086). However, with regard to the number of medications, statistically significant differences are observed when comparing both study groups (adequate-ML and inadequate-ML; *P* < 0.001; OR = 0.345; 95% CI: 0.225–0.530). A higher adequate literacy rate was observed for clients who did not take medicine (49%) than for those taking some form of medication, 1–4 medicines (31.8% and ≥ 5 medicines (7.1%) *P <* 0.001; OR = 0.345; 95% CI: 0.225–0.530). However, no significant statistical difference was found with respect to reading the information leaflets (*P* = 0.180).

Finally, analysis of the mean number of correct responses to the MedLitRxSE tool (14 questions) reflecting total medical literacy found an average of 10.30 ± 3.41 correct answers (mean points ± SD; range: 0–14 points) in the total population analyzed (Table 1). No difference was observed related with gender, both obtaining a similar and high percentage of correct answers (*P* = 0.075).

By contrast, a gradual decrease in the number of correct answers was observed when increasing age (*P* < 0.001). A gradual decrease in the number of correct answers was also observed as the number of medicines consumed increased (*P <* 0.001). A secondary school level of education was related with the highest number of correct answers (12.67 ± 1.46, mean ± SD). No significant differences (*P* = 0.083) were associated with pharmacy location (rural or urban). Finally, a lower number of correct responses was observed in users that never read information leaflets compared with the clients who read always or sometimes the leaflet (*P* = 0.004).

### Analysis of documental literacy

Analysis of the 10 questions that reflect the extent of documentary literacy in MedLitRxSEs showed an average number of correct answers of 7.06 ± 2.51 (mean ± SD) in the total population analyzed (Table 1). Statistically significant differences were found between men and women (*P* = 0.008; OR = 1.79; 95% CI (1.17–2.73)) as regards the number of correct responses but this association was not confirmed when multivariable analysis was applied (*P* = 0.090; OR = 1.54; 95% CI (0.93–2.56); Table 2). The number of correct answers was lower in individuals over 65 years of age compared with the rest of ages analyzed (*P <* 0.001). On the other hand, a greater number of correct answers was observed in individuals with a minimum of secondary education (8.79 ± 1.28, mean ± SD) than in others groups. This association were confirmed when multivariable logistic regression analysis was applied (*P* < 0.0001, OR = 12.31; 95% CI (6.33–23.92); Table 2). No association was observed between rural and urban area pharmacies (*P* = 0.069). Documentary literacy was higher in clients who did not take medicines compared with those that did (*P* < 0.001, OR = 2.93; 95% CI (1.92–4.48). Patients with chronic medication use was also considered a factor associated with ML (*P <* 0.001, OR = 2.93; 95% CI (1.92–4.48), however this observation was not confirmed by multivariate analysis. However, best level of documentary literacy was recorded in pharmacy customers who always read the leaflets.

**Table 2:** Univariate and multivariate logistic regression of the MedLitRxSE, document and numerical literacy based on adequate ML

Univariate logistic regression		P	OR	95% CI		Multivariate logistic regression*		Wald	P	OR	95% CI	
				Lower	Upper						Lower	Upper
**MedLitR**_**x**_**SE**						**MedLitR**_**x**_**SE**						
**Education**						**Education**						
	**Primary or no study**		**1**				**Primary or no study**			1		
	**Secondary**	**< 0.0001**	10.42	5.27	20.62		**Secondary**	45.37	**< 0.0001**	**10.42**	**5.27**	**20.61**
	**University**	**< 0.0001**	25.95	12.66	53.17		**University**	79.11	**< 0.0001**	**25.94**	**12.66**	**53.16**
**No Chronic medication**		**< 0.001**	**2.89**	**1.88**	**4.45**							
**Document literacy**						**Document literacy**						
**Gender**	**Male**	**0.007**	1.79	1.17	2.73	**Gender**	**Male**	**2.87**	**0.09**	**1.54**	**0.93**	**256**
	**Female**		1				**Female**			1		
**Education**						**Education**						
	**Primary or no study**		1				**Primary or no study**			1		
	**Secondary**	**< 0.0001**	12.65	6.52	24.55		**Secondary**	54.82	**< 0.0001**	12.31	6.33	23.92
	**University**	**< 0.0001**	27.27	13.46	55.26		**University**	82.39	**< 0.0001**	26.56	13.08	53.92
**Chronic medication**		**< 0.001**	**2.93**	**1.92**	**4.48**							
**Numeracy literacy**						**Numeracy literacy**						
**Age**	**< 35**	**< 0.0001**	16.59	7.99	34.45	**Age**	**< 35**	10.48	**0.01**	4.22	1.76	10.11
**(years)**	**35–50**	**< 0.0001**	9.01	4.64	17.50	**(years)**	**35–50**	11.72	**0.01**	3.86	1.78	8.36
	**51–65**	**0.004**	3.79	1.96	7.32		**51–65**	8.77	**0.03**	3.08	1.46	6.49
	**> 65**		1				**> 65**			1		
**Education**						**Education**						
	**Primary or no study**		1				**Primary or no study**			1		
	**Secondary**	**< 0.0001**	8.52	5.04	14.39		**Secondary**	30.78	**< 0.0001**	5.35	2.96	9.69
	**University**	**< 0.0001**	24.94	12.18	51.05		**University**	51.43	**< 0.0001**	16.98	7.79	36.55
**No Chronic medication**		**< 0.001**	**4.12**	**2.61**	**6.48**							

**Fig. 2:**
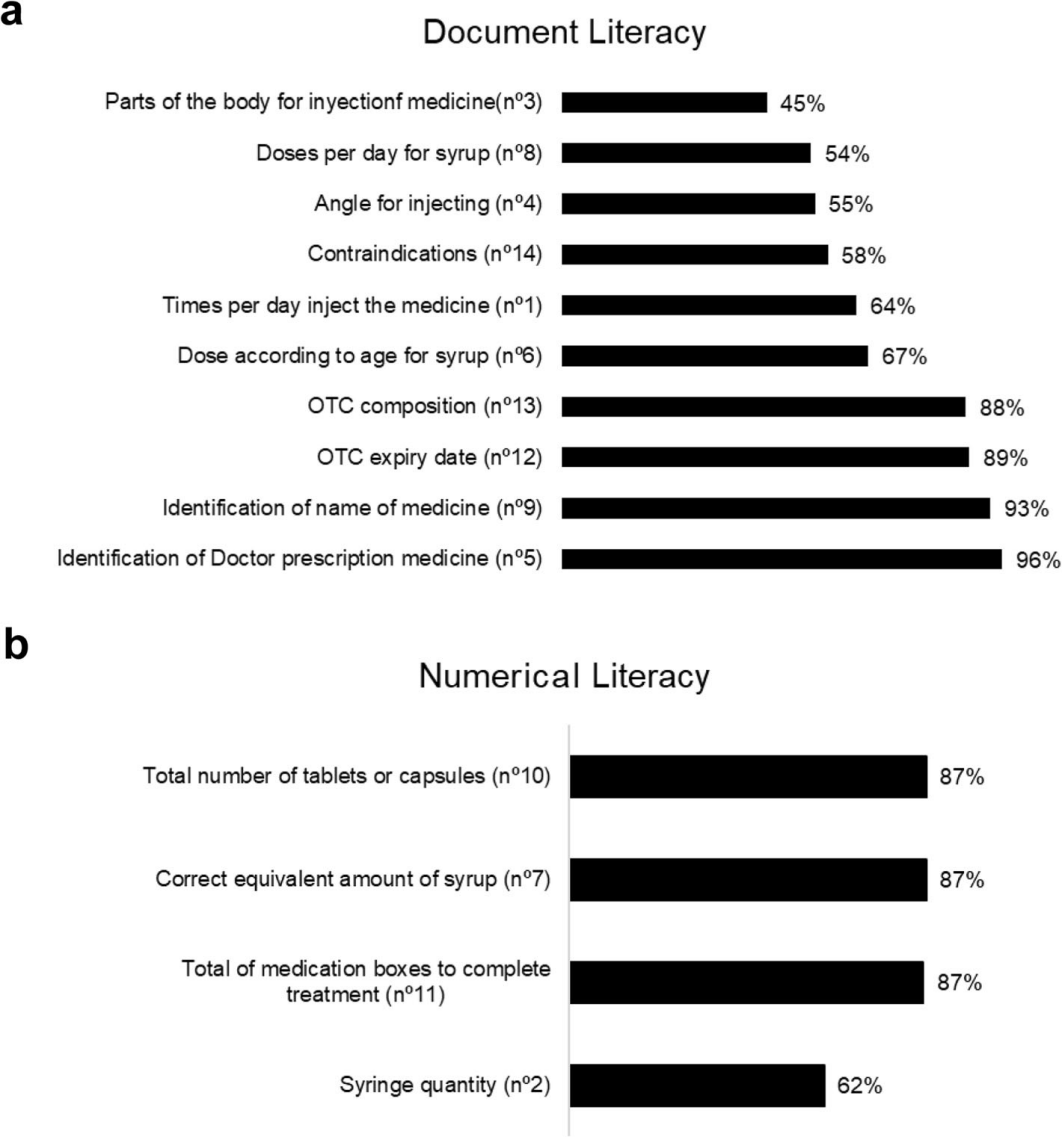
Frequency of correct answers in the MedLiTRxSE tool. (**a**) Frequency of correct answers related to documental literacy (**b**) Frequency of correct answers related to numerical literacy

Finally, the different scenarios in the MedLiTRxSE tool for documental-ML were also analyzed (Fig. 2a). The greatest difficulty to answer adequately involved identifying the parts of the body to inject medicine (45%, scenario #3) and the daily doses per day of syrup medicines (54%; scenario #8). By contrast, there was a high frequency of correct answers (96%) for identifying medicines that could only be prescribed by a doctor (scenario #5) for identifying the name of a medicine (93%; scenario #9).

### Analysis of numerical literacy

Analysis of the four questions that reflect the numerical literacy in MedLitRxSEs found a mean of 3.23 ± 1.09 (mean ± DS) correctly answered questions by the total cohort (Table 1). As regards the sociodemographic characteristics analysed, numerical literacy did not differ with gender (*P* = 0.268) or the location of the community pharmacy (*P* = 0.154), but statistically significant differences were found as a function of age (*P* < 0.001), numerical literacy decreasing as the patient’s age increased and also in terms of education level (*P <* 0.001). The patient’s age was confirmed by multivariable logistic regression analysis (*P* < 0.0001; Table 2).

Individuals with primary studies or no formal studies showed a low level of numerical literacy (1.86 ± 1.17; mean ± DS) compared with individuals with a university education (3.88 ± 0.35; mean ± SD). These associations were confirmed through multivariable logistic regression analysis (*P <* 0.0001; Table 2). Differences were also observed between individuals who never read the information leaflet and those who always read it (*P* = 0.002; Table 1).

When the frequency of correct answers in the MedLiTRxSE tool for numerical literacy were also analyzed (Fig. 2b), the numerical calculation of units of injectable medicine for diabetes (scenario #2) was the problem that presented the lowest frequency of correct answers (62%) compared with the rest of the questions whose frequency of success was 87% in all of them.

## Discussion

In this study the ML of clients acquiring prescription and non-prescription medicines in community pharmacies was analyzed using MedlitRxSE questionnaire in order to know the medication literacy of users, with special emphasis on the chronically ill with the future purpose of designing new strategies that allow the pharmacist and health personnel to prevent and reduce the errors in taking medicines and thus avoid the undesirable effects of any misuse.

Pharmacists, as other health care providers, sometimes use terminology that their patients find difficult to understand. Moreover, in contrast to comprehensive (verbal) patient therapy, it is not unusual for health care providers, including pharmacists, to rely heavily on written patient education materials such as the leaflets that usually accompany medicines [20].

However, there are differences between what patients really understand and what health care providers, including pharmacists, expect or believe them to know. Patients with low reading ability have been found to be substantially less likely to understand and remember drug advice, and more likely to have trouble knowing exactly what most health professionals recommend [3, 19, 20].

The incorrect use of medication is a major problem, not only because it diminishes the effectiveness of medicines, but also because of the high frequency of problems associated with their misuse [25]. In this study, MedLitRxSE proved to be an effective and easy-to-use tool to assess the literacy of patients, and thus play an important role in ensuring patient safety and adherence to the instructions on how to use medicines provided by community pharmacies. Knowledge and improvement of ML could help reduce non-adherence to treatments, enabling patients to participate mor fully in their medication therapy [26].

Our result showed that only 34% of community pharmacy clients can be considered to have an adequate level of ML, which is similar to the findings of Sauceda et al. [13] in a population of 181 English and Spanish speaking patients in health centers and in the general population. Our results also suggest that ML decreases as the age of clients increase, as mentioned by Lee YM et al. [27]. Another study showed that patients with limited health literacy have a significantly low understanding of the instructions on the label of medicine containers, and therefore a higher risk of having problems related to the medication [28].

A predictive factor of adequate total, documental and numerical literacy was the educational level of participants. Moreover, in the case of numeracy, a younger age was also seen to be a predictive factor. The same factors associated with ML are also mentioned in the literature as predictors of health literacy [29, 30]. In a study conducted by Osborn et al. [29] with 205 patients, health literacy was measured using the Rapid Estimate of Adult Literacy in Medicine (REALM) and the Wide Range Achievement Test, 3rd Edition (WRAT-3). The authors found that both numeracy and health literacy improved as the level of studies and income increased. Okamoto et al. [31] measured numeracy-ML in 300 people aged between 20 and 69 years using Lipkus and Schwartz scales. The first scale identified 46.33% of participants as having a low level of literacy, while the Schwartz scale identified 39.67% of participants as having a low literacy level. Men who had had a university education had the highest scores, while as age increased, the score decreased. Income did not have any effect on the results obtained for numeracy. In another study, using the short test on functional health literacy (S-TOFHLA) in a study of patients from different pharmacies in the United States, Backes and Kuo [32] observed that patients in general did not have an adequate functional level of health literacy, often did not remember the name of their treatment compared with patients considered to have a correct functional level of health literacy. The same occurred with remembering the correct dose and the frequency with which the medicine should be taken.

In view of our results, the advantages offered by using MedLitRxSE tool to analyze ML rather other health literacy questionnaires is that it allows the needs of clients, more specifically in the pharmaceutical environment, to be quickly and efficiently detected, thus improving pharmaceutical care and management of medications by the patient.

The results pointed to a decrease in adequate medication literacy as the number of medications consumed by the patient increases. However, in a study carried out by Lyles et al. [33] no association was found between health literacy and medication adherence or between health literacy and polypharmacy. Bauer et al. [34] studied the relationship between health literacy and adherence to following treatment correctly in a large cohort of patients treated with antidepressants during a 4 year follow-up and observed that 72% of patients could be classified as having limited health literacy, these patients show little adherence to medications compared to patients without such limitations.

Our results show that the scenario with the most problems of interpretation on the part of clients was that related with identifying the part of the body to inject a medicine. To solve this problem, it would be interesting to add illustrations to help patients with low levels of literacy to improve their understanding of how medicines should be used. Indeed, a study showed that this type of illustration could reduce errors both in the dosage of the medicine and resolve doubts about the part of the body where medication should be applied, at the same time increasing the degree of satisfaction with the care received in the community pharmacy in patients with low ML showed [35, 36]. However, another study carried out with that the use of illustrations did not reinforce the information received only in written form [37]. However, the illustrations must be clear because it has also been shown that the illustrations themselves may be a source of errors that result in improper administration of the medication [38]. One alternative to the use of written information and illustrations could be providing the information about medication in audio format. This has been used for treating patients with a low level of health literacy with statins, and was seen to increase knowledge about the medication and patient satisfaction compared with those who received the usual information materials [39].

In terms of numeracy-ML, 57% answered the 4 questions correctly, unlike in the study carried out by Osborn et al. [29], in which only 38.24% of participants were adjudged to have an adequate level of numeracy-ML. Participants in our study made mistakes most frequently in the question related with the medication dose required, which can lead to overdosing. It should be noted that users with medium level or higher education had a significantly higher level of total ML than users without formal education or those with only primary education. In a study conducted in 7278 community pharmacy patients throughout Spain, Romero-Sanchez et al. [40] observed that uneducated patients had a higher risk of not understanding the information on the medication than patients with primary, secondary or university level studies.

The more frequently patients read information leaflets, the higher their score for total, numeracy and document-ML. In this sense, some authors have suggested that the habit of frequent reading is a powerful tool for improving health literacy [41].

It should be noted that the MedlitRxSE questionnaire has not been used in a wide variety of situations. Although these cut-off points are due to expert criteria, a certain subjective charge cannot be denied as a limitation. To our knowledge, there are few instruments exclusively dedicated to measuring ML. Therefore, one of the problems with discussing the data is that it is difficult to compare our results with other studies specifically referring to ML, whether or not MedLitRxSE was used or other similar tools.

In addition, the survey used in our research to measure ML does not measure the communication skills of patients, which forms part of health literacy and is essential for interaction with health professionals, with the health care system and for understanding the warnings related to medication and health [42].

On the other hand, the original instrument was developed in the United States with a Spanish-speaking sample there. In our study, the instrument was used in a completely different context, being a limitation of our study. In the original developmental study, the instrument was validated showing that it was related to the Short Test of Functional Health Literacy in Adults (S-TOFHLA), a measure of health literacy with only a few questions that directly address medication management. Therefore, MedLitRxSE has been validated as a measure of general health literacy. It may have content validity due to the nature of the questions, however in our study we have taken into account that the measure of knowledge about drugs is only valid in the sense that it includes questions about medication management.

## Conclusions

In conclusion, the MedlitRxSE questionnaire can be considered a useful tool for measuring ML effectively and rapidly in the community pharmacy. It is a structured, easy-to-complete tool for participants and useful for evaluating the knowledge, abilities and skills necessary for managing personal medication. Their use in the present study found that community pharmacy clients over the age of 51 and those with lower levels of education have a significantly lower LD level than younger age groups and those with higher levels of education. Knowledge of patient ML would help improve communication and contribute to increasing user knowledge and understanding of their illnesses and the pharmacotherapy involved. Such knowledge would increase adherence to treatment, and the clinical outcome and safety of the same. This should encourage pharmacists as health professionals to adopt strategies and initiatives that will improve the skills and abilities necessary for managing medication, especially in the population groups mentioned, for example, by encouraging patients to read information leaflets.

On the other hand, it should be noted that this analysis has allowed us to know the LA of the patients with different sociodemographic characteristics in the community pharmacies analyzed. Currently, pharmacists have access to the electronic prescription of all chronic patients who come to pick up their medication at the pharmacy. This study allows pharmacists to detect and know which group and which sociodemographic characteristics of their clients need more and more personalized attention, with more detailed explanations when drugs are dispensed, with the intention of reducing errors in taking medications.

## Supplementary Information

**Additional file 1: Table S1.** Scenarios established in the MedLitRxSE tool for Spanish drugs, with their relevant questions and their relationship with literacy and documentary literacy.

## Data Availability

The datasets used during the current study are available from the corresponding author on reasonable request.

## Abbreviations

CI: Confidence intervals
I2: I-square
LCL: Lower confidence level
MedLitRxSE: Medication Literacy Assessment
ML: Medication literacy
OR: Odds ratios
REALM: Rapid Estimate of Adult Literacy in Medicine
SD: Standard deviation
S-TOFHLA: Short test on functional health literacy
UCL: Upper confidence level
WRAT-3: Wide Range Achievement Test, 3rd Edition
