# Supplementary material for: Age and education as factors associated with medication literacy: a community pharmacy perspective
Source: BMC Geriatr. 2020 Nov 25;20:501. doi: 10.1186/s12877-020-01881-5 (PMC7687724; doi:10.1186/s12877-020-01881-5)
Supplement: Supplementary file 1 — Additional file 1: Table S1. Scenarios established in the MedLitRxSE tool for Spanish drugs, with their relevant questions and their relationship with literacy and documentary literacy. [file 12877_2020_1881_MOESM1_ESM.doc]

| **Table S1.** Scenarios established in the MedLitRxSE tool for Spanish drugs, with their relevant questions and their relationship with literacy and documentary literacy. | | |
| --- | --- | --- |
| **Nº** | **MedLiTRxSE*** | **Type of medication**  **literacy** |
|  | **Case Scenario #1: Injectable Medicine for Diabetes** |  |
| 1 | Identification the number of times a day inject the medicine | Document |
| 2 | Identification of the amount of medicine to put in the syringe | Numeracy |
| 3 | Identify/explain parts of the body for injectable medicine | Document |
| 4 | Identify the correct angle to put this subcutaneous injection | Document |
| 5 | Identification of the doctor who preinscribes the medicine | Document |
|  |  |  |
|  | **Case Scenario #2: Children’s dosing of acetaminophen** |  |
| 6 | Identification of the amount of syrup | Document |
| 7 | Identification of the correct equivalent amount of syrup | Numeracy |
| 8 | Identification of dose per day of medicine | Document |
|  |  |  |
|  | **Case Scenario #3: Spanish drug product** |  |
| 9 | Identification of the name of the medicine | Document |
| 10 | Identification of the number of tablets or capsules of medication in total you need to take to treat the disease | Numeracy |
| 11 | Identification of the number of medication boxes you would need to complete a treatment. | Numeracy |
|  |  |  |
|  | **Case Scenario #4: OTC and Prescription with auxiliary label** |  |
| 12 | Identification of the correct expiration date on OTC product | Document |
| 13 | Identification of the active ingredients | Document |
| 14 | Identification of the warning about medication side effects | Document |
